# Excellent local control and survival after postoperative or definitive radiation therapy for sarcomas of the head and neck

**DOI:** 10.1186/s13014-015-0449-x

**Published:** 2015-07-10

**Authors:** Claudia Andrä, Josefine Rauch, Minglun Li, Ute Ganswindt, Claus Belka, Ladan Saleh-Ebrahimi, Hendrik Ballhausen, Silke Birgit Nachbichler, Falk Roeder

**Affiliations:** Department of Radiation Oncology, University Hospital of LMU Munich, Marchioninistr, 15 81377 Munich, Germany; Department of Molecular Radiation Oncology, German Cancer Research Center (DKFZ), Heidelberg, Germany

## Abstract

**Background:**

To report our results with postoperative or definitive radiation therapy in head and neck sarcomas.

**Methods:**

We performed a retrospective analysis of 26 patients suffering from head and neck sarcomas, who received postoperative or definitive radiation therapy between 2003 and 2012. Median age was 64 years (19–88) and 69 % were male. Tumor locations were skull (including skin) in 31 %, paranasal sinus/orbita in 27 % and neck (including pharynx/larynx) in 42 %. Median tumor size was 4.6 cm (1-12 cm). 22 patients (85 %) presented in primary situation. Stage at presentation (UICC 7^th^ for soft tissue sarcomas) was as follows: Ia:4 %, IIa:50 %, IIb:15 %, III:31 %. All except one patient suffered from high grade lesions (G2/3 FNCLCC), predominantly angiosarcoma (35 %), MFH (19 %) and synovial sarcoma (15 %). Surgery was performed in 21 pts (81 %), resulting in free margins in 10 (38 %), microscopically positive margins in 6 (23 %) and gross residual disease in 5 (19 %). Median dose to the primary tumor region was 66Gy (45-72Gy) in conventional fractionation, using 3D-CRT in 65 %, IMRT in 27 % and electrons in 8 %. 50 % of the patients also received sequential chemotherapy.

**Results:**

Median follow up was 39 months (8–136). We observed three local recurrences, transferring into estimated 3- and 5-year local control rates of 86 %. One additional patient failed distantly, resulting in 3- and 5-year freedom from treatment failure rates of 82 %. Four patients have deceased, transferring into 3- and 5-year overall survival rates of 88 % and 82 %, respectively. Only two of the four deaths were sarcoma related. Maximum acute toxicity (CTCAE 3.0) was grade 1 in 27 % of the patients, grade 2 in 50 % and grade 3 in 23 %. Severe acute toxicity was mainly represented by mucositis and dysphagia. Maximum late toxicity was grade 1 in 31 %, grade 2 in 15 % and grade 3 in 19 % of the patients. Severe late toxicity included skin ulceration (n = 1), dysphagia with persistent tube dependency (n = 1), persistent sinusitis (n = 1) and hearing loss (n = 2).

**Conclusion:**

Excellent local control and overall survival rates can be achieved with postoperative or definitive radiation therapy with acceptable acute and late toxicities in patients suffering from sarcomas of the head and neck region.

## Background

Soft tissue sarcoma of the head and neck is a very rare disease, given the fact that soft tissue sarcomas represent less than 1 % of all malignancies in adults [1] and only 5–10 % of them are located in the head and neck region [2]. Because of this rarity, evidence from randomized trials or prospective studies is almost absent [3]. Treatment recommendations are mainly based on data from trials performed in extremity sarcomas [4], although achievement of local control seems more critical with regard to overall survival [3, 5] in the head and neck region than in other sites. Surgery remains the cornerstone of curative intent treatment [5], however wide margins are often difficult to achieve due to the proximity of crucial structures or inacceptable functional outcome. Therefore postoperative radiation therapy has been advocated by some authors [3, 6] based on the clear evidence for improved local control with this approach compared to surgery alone in extremity sarcomas [7]. In cases without the possibility of gross total resection, definitive high dose radiation therapy seems to be the best local treatment option [8]. However, no generally accepted standard for target volume definition and dose prescription in this particular entity exists. Here we present our experience with postoperative or definitive radiation therapy in head and neck sarcoma cases.

## Methods

We performed a retrospective analysis of 26 patients suffering from head and neck soft tissue sarcomas without distant spread, who were treated with postoperative or definitive radiation therapy at our institution between 2003 and 2012. Patients with dermatofibrosarcoma protuberans (DFSP) or desmoid tumors were excluded. Median age was 64 years and 69 % of the patients were male. 22 patients (85 %) presented in primary situation while four had already recurrent tumors. Tumors were located in the skull in 31 %, paranasal sinus/orbita in 27 % and neck (including pharynx/larynx) in 42 % of the patients. Median tumor size was 4.6 cm. All except one patient suffered from high grade lesions (G2/3 according to FNCLCC), predominantly angiosarcoma (35 %). Clinical stage at presentation (according to UICC 7^th^ edition for soft tissue sarcomas) was Ia in 4 %, IIa in 50 %, IIb in 15 % and III in 31 % of the patients. For detailed patients characteristics see Table 1.

**Table 1:** Patient and treatment characteristics

Table 1	n	%		n	%
Gender			Age		
Male	18	69	Median	64 years	
Female	8	31	Range	19–88 years	
Situation			Size		
Primary	22	85	Median	4.6 cm	
Recurrent	4	15	Range	1–12 cm	
Location			Grading		
Skull*	8	31	1	1	4
Sinus/orbit	7	27	2	8	31
Neck‘	11	42	3	17	65
Histology			T stage		
Angiosarcoma	9	35	1a	4	15
MFH/undiff. pleo.	5	19	1b	10	38
Synovial	4	15	2b	12	46
Other	8	31			
N stage			CHT		
N0	24	92	Yes	13	50
N1	2	8	None	13	50
Clinical stage			Surgery		
Ia	1	4	R0	10	38
IIa	13	50	R1	6	23
IIb	4	15	R2	5	19
III	8	31	None	5	19
RT dose (PT)			ENI		
Median	66 Gy		Yes	14	54
Range	45–72 Gy		No	12	46
RT technique					
3D-CRT	17	65			
IMRT	7	27			
Electrons	2	8			

*: includes skin, ‘: includes larynx/pharynx, MFH: malignant fibrous histiocytoma, undiff. pleo.: undifferentiated pleomorphic sarcoma, RT: radiation therapy, PT: primary tumor region, 3D-CRT: 3d-conformal radiation therapy, IMRT: intensity-modulated radiation therapy, cm: centimeter, CHT: chemotherapy, ENI: elective nodal irradiation (ipsilateral cervical nodes)

Surgery was performed in 21 (81 %) patients and resulted in free margins in 10 (38 %), microscopically positive margins in 6 (23 %) and gross residual disease in 5 (19 %) patients. 5 (19 %) patients were judged primarily unresectable. Radiation therapy was performed either postoperatively or definitively. Due to the long time period covered by this study, target delineation, radiation technique and dose varied to some extent. Usually patients were treated in supine position, using a thermoplast head mask and multiple field techniques (3D-conformal RT or IMRT). The initial PTV usually included the surgical bed/gross tumor volume with a safety margin of 2–3 cm and was irradiated up to 50–50.4 Gy in conventional fractionation (1.8–2.0 Gy single dose). In patients with node positive disease, after ipsilateral neck dissection or histologies known for increased risk of nodal spread (e.g. synovial sarcoma, angiosarcoma), the ipsilateral cervical lymph nodes were included into the initial PTV. The boost PTV usually included the surgical bed/gross tumor volume with a safety margin of 1–2 cm and received 10–20 Gy according to resection margin. Margins could be reduced at anatomical borders like uninvolved bony structures or in case of directly adjacent organs at risk with low radiation tolerance at the discretion of the treating radiation oncologist. Half of the patient additionally received pre- or postoperative sequential chemotherapy (mainly doxorubicin and/or ifosfamide) at the discretion of the treating medical oncologist. For detailed treatment characteristics see Table 1.

Regular follow-up took place at our institution or at the referring center and included at least clinical examination, CT or MRI of the head and neck area and scoring of toxicity. In case of evidence for local or distant spread, additional tests were performed at the discretion of the treating physician to confirm or rule out treatment failure. Local control (LC) was defined as absence of tumor regrowth in the head and neck area. Freedom from treatment failure (FFTF) was defined as absence of local or distant failure. All time to event data was calculated from the first date of radiation treatment according to the Kaplan-Meier method. Acute and late toxicities were scored according to CTCAEV3.0. No subgroup analyses were performed due to the small sample size. The study was in accordance to the declaration of Helsinki in its latest version.

## Results

**Fig. 1:**
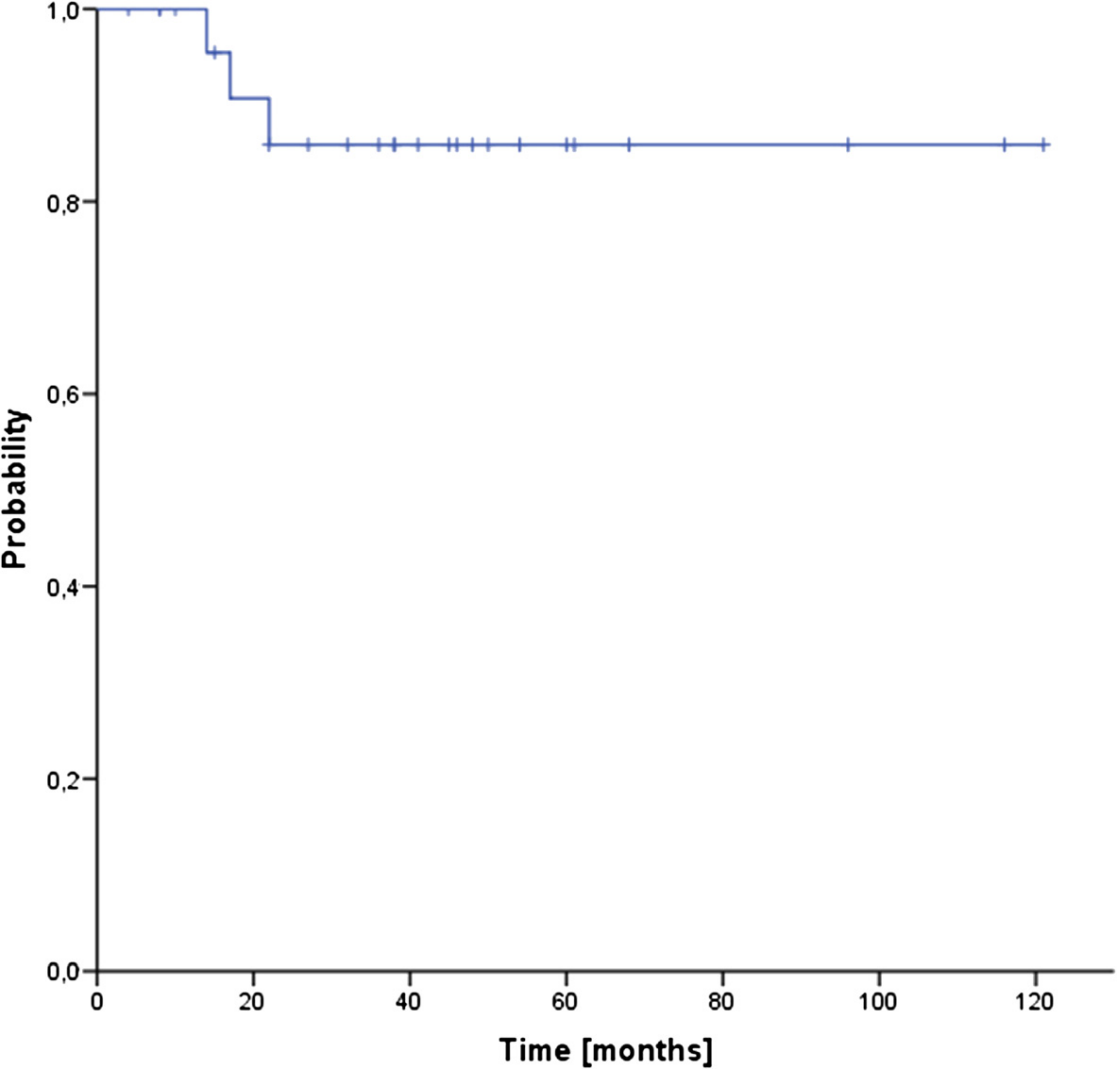
Local control (LC)

**Fig. 2:**
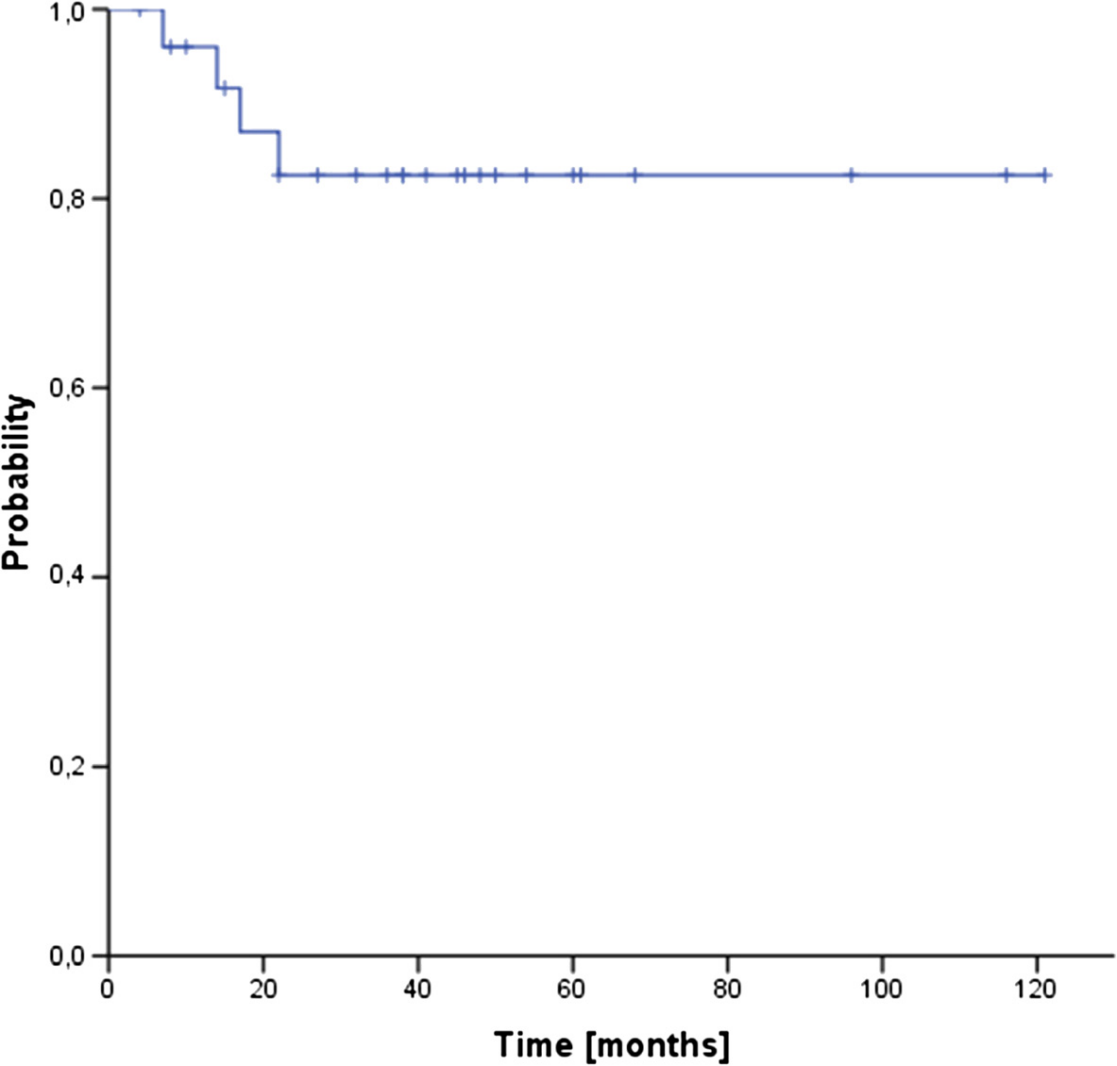
Freedom from treatment failure (FFTF)

**Fig. 3:**
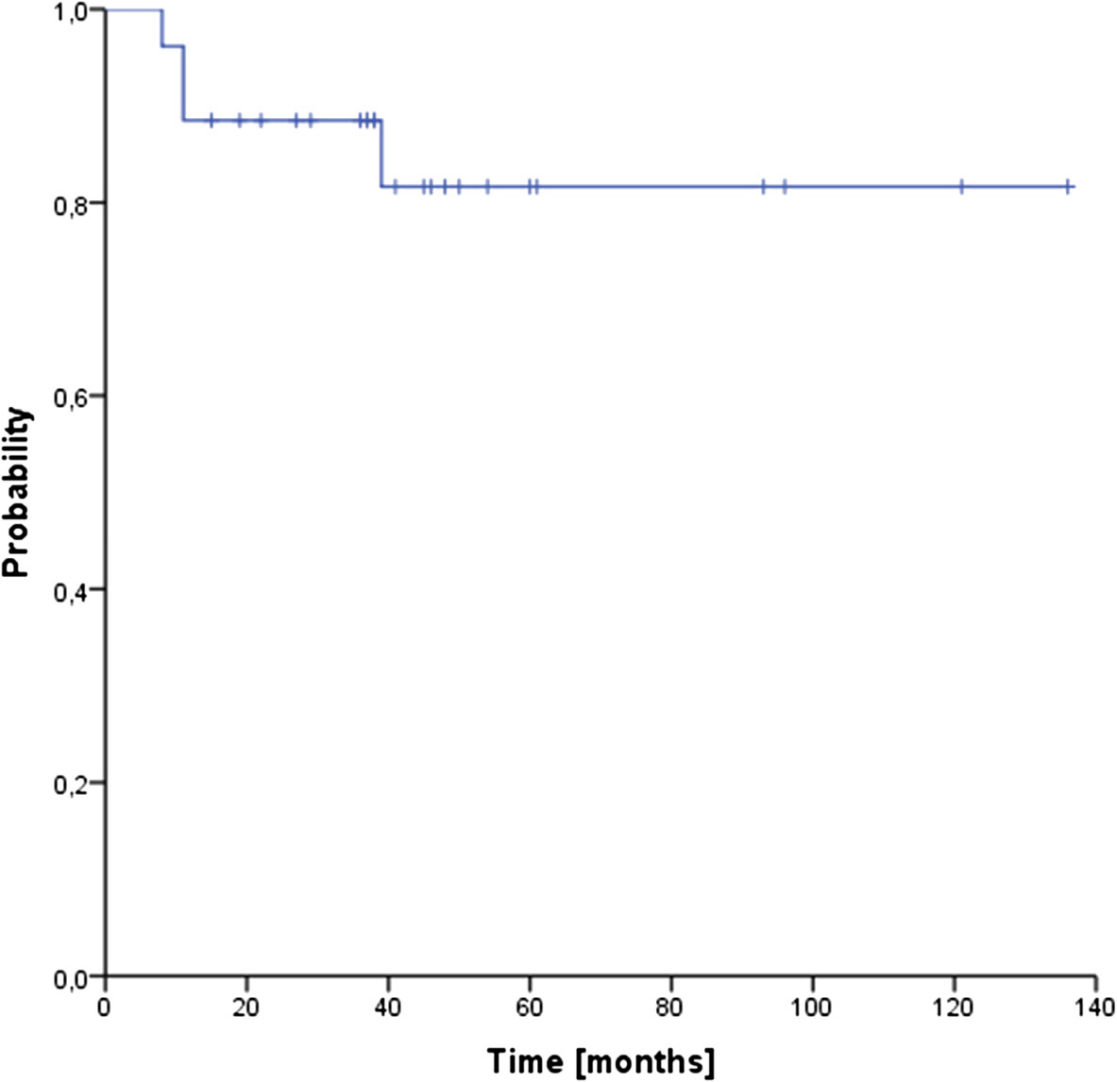
Overall survival (OS)

Median follow up for the entire cohort was 39 months (range 8–136 months). Three patients (12 %) developed local recurrences after 14, 17 and 22 months, transferring into estimated 3- and 5-year local control rates of 86 % (see Fig. 1). One additional patient failed distantly (at multiple sites) after 10 months, resulting in 3- and 5-year freedom from treatment failure rates (FFTF) of 82 % (see Fig. 2). Four patients (15 %) have deceased, transferring into 3- and 5-year overall survival rates of 88 % and 82 %, respectively (see Fig. 3). Only two of the four deaths were sarcoma related.

Acute radiation related toxicities consisted mainly of dermatitis, mucositis and dysphagia, but were generally mild. The maximum acute toxicity was grade 1 in 27 %, grade 2 in 50 % and grade 3 in 23 % of the patients. No grade 4/5 acute toxicities were observed. For detailed acute side effects see Table 2. Late radiation related toxicities were even less pronounced and did not show a distinct pattern. Maximum late toxicity was grade 1 in 31 %, grade 2 in 15 % and grade 3 in 19 % of the patients. Severe late side effects included skin ulceration (1), dysphagia with persistent tube dependency (1), persistent sinusitis (1) and hearing loss (2 patients). No grade 4/5 late toxicities have been observed. For detailed late toxicities see Table 3.

**Table 2:** Acute radiation-related toxicities, multiple toxicities in same patient possible

Table 2	All grades		Grade 3	
	n	%	n	%
Skin	24	92	3	12
Mucositis	16	62	2	8
Dysphagia	16	62	4	15
Otitis/Hearing	3	12	1	4
Hoarseness	6	23		
Nausea	3	12		
Dry eye	3	12		
Epistaxis	2	8		
Leukopenia	10	39	1	4
Anemia	15	57		
Thrombopenia	2	8

**Table 3:** Late radiation-induced toxicities, multiple toxicities in same patient possible

Table 3	All grades		Grade 3	
	n	%	n	%
Skin	5	19	1	4
Lymph edema	3	12		
Dysphagia	2	8	1	4
Xerostomia	8	31		
Loss of taste	4	15		
Hearing loss	3	12	2	8
Sinusitis	1	4	1	4
Hypothyreosis	1	4		
Hoarseness	1	4		
Dry eye	1	4		
Skin infection*	1	4	1	4
Trismus	1	4		

*Probably not related to radiation

## Discussion

Soft tissue sarcomas of the head and neck are a very rare entity, usually treated with surgery and/or radiation therapy [6]. In our current series, we evaluated 26 patients who had received postoperative or definitive radiation therapy to a median dose of 66 Gy and found an encouraging 5-year-LC rate of 86 % and a 5-year-OS rate of 82 %. These results seem to compare favourably with many other series, which reported 5-year LC rates of 41 %-83 % [2, 5, 9–18] and 5-year survival rates of 56 %-75 % [2, 5, 9–18]. However, care must be taken when interpreting and comparing different series of head and neck sarcomas. Most available data is based on retrospective analyses including small numbers of patients and covering long time periods. Variations in pathologic subsite, site of tumor involvement, local tumor extent, percentage of gross total resections, percentage of irradiated patients and histologic grade are all factors with impact on outcome [6]. For example, Mattavelli et al. [5] reported on 167 patients of whom the vast majority received gross total resection (94 %) without adjuvant therapy (66 %). They reported encouraging 10-year local recurrence and disease-specific mortality rates of 19 % and 26 %, respectively. However, 35 % of the included patients suffered from dermatofibrosarcoma protuberans (DFSP), which minimally contributed to the occurrence of any events [5]. If those patients were excluded, 10-year LR and DSM rates increased to 27 % and 39 %, respectively. Le et al. [15] reported a series of 65 patients, of whom less than half received gross total resection but were postoperatively irradiated in the majority of cases (78 %). In the 54 patients treated with curative intent, they achieved 5-year local control and overall survival rates of 66 % and 64 %, respectively. Willers et al. [10] analyzed 57 patients, who were treated with radiation therapy (with or without surgery) and found 5-year local control rates of 60 % and 66 %, respectively. After exclusion of angiosarcomas, local control and survival were improved to 69 % and 83 %. Recently, Trifiletti et al. [18] reported a small series of 28 patients with mature follow up (median 11 years) of whom all received gross total resection and adjuvant radiation therapy and found very encouraging 5-year LC and OS rates of 83 % and 75 %, respectively. Given the inhomogeneous patient cohorts, it is difficult to assess the influence of treatment related factors like margin status or the efficacy of adjuvant radiation therapy. In most series, favourable, small, low grade tumors tended to be treated with surgery alone, whereas unfavourable tumors with incomplete resections were more likely to receive additional radiation therapy [6]. However, there is a strong rationale to add radiation therapy at least in the majority of patients: As known from randomized trials and large registry studies, additional radiation therapy undoubtly increases local control after wide excision in extremity sarcomas [7, 19, 20]. The absolute gain for the addition of radiation therapy thereby increases with grade and narrowness of the surgical margin [20]. As wide surgical margins are often not achievable in head and neck cases due to the proximity of vital structures and even gross residual disease will be present in a substantial proportion of patients after surgery [6], additional radiation therapy should theoretically result in even more pronounced improvements in local control as in extremity sarcomas. Consistently with that assumption, most series reporting subgroup analyses showed at least improved local control rates for the combination of surgery and radiation compared to surgery alone, even considering an imbalance of prognostic factors in favour of the surgery only group [13, 15, 16]. For example Le et al. [15] reported 5-year local control rates of 59 % for surgery alone and 77 % for surgery and RT, although the combined group included larger tumors and more incomplete resections. Eeles et al. [16] observed 5-year local control rates of 40 % for surgery alone and 60 % for the combined treatment and described combination treatment as a significant positive factor in multivariate analysis for local control. Tran et al. [13] compared 94 patients treated with surgery with or without adjuvant radiation at UCLA and observed a significant difference in local control rate (52 % vs 90 %) in favour of adjuvant radiation. They further analyzed the group according to margin status and found the most pronounced difference in patients with positive margins.

The achievement of local control is of crucial interest in head and neck sarcomas because of the different pattern of relapse compared to extremity sarcomas. Similarly to the retroperitoneal space [21], many series showed that in head and neck sarcomas local recurrence occurs more often than distant relapse and represents the major cause of death because salvage surgery is often limited [3]. For example in the series of Mattavelli et al. [5], 25 of the 35 locally recurrent patients died as a consequence of their relapse and the authors concluded that more patients were lost due to local than due to distant progression. Eeles et al. [16] described 46 sarcoma related deaths in their series, of whom 30 were caused by local and only 16 by distant progression. Le Vay et al. [9] described that 68 % of sarcoma related deaths were caused by uncontrolled local relapse. Willers et al. [10] found an even higher percentage of 74 % in their series. Correspondingly, the rate of distant failures seems lower than in extremity sarcomas according to most series. Kraus et al. [6] reviewed the literature and found rates of 0–9 % for distant metastases at presentation and 9–31 % for subsequent distant failure after treatment of initially locoregionally confined disease.

Because of the rarity of head and neck sarcomas, no generally accepted dose and target volume concept exists for adjuvant or definitive radiation therapy. Usually the treatment of the tumor bed after surgery with generous margins is recommended based on the experience from extremity sarcomas. Dose concepts also follow the recommendations for extremity tumors, but are often difficult to achieve due to adjacent radiosensitive organs at risk. In general 60–70 Gy are recommended by most authors depending on margin status [6]. In our series we used doses of 45–72 Gy with a median dose of 66 Gy. Attempted doses were 60–72 Gy according to margin status. With this dose concept we observed acceptable rate of severe acute (grade 3: 23 %) and late (grade 3: 19 %) side effects. No grade 4/5 toxicities were found. Based on the experience in extremity sarcomas, growing evidence for a dose-effect relationship exists regarding local control but also toxicity [22]. For example, Zagars et al. [23] found improved local control rates after gross complete resection with doses of 64–68 Gy compared to 60 Gy. Fein et al. [24] demonstrated improved local control rates if doses of 65 Gy or more were used. For gross residual disease, even higher doses have to be attempted to achieve durable local control at least in a substantial proportion of patients. For example, Tepper et al. [25] found a significantly improved local control rate with doses of more than 64 Gy in a series of unresectable soft tissue sarcomas. Slater et al. [26] described longer duration of local control after doses exceeding 65 Gy and Kepka et al. [8] reported significantly improved local control, disease-free survival and overall survival rates in unresectable soft tissue sarcoma patients treated with doses of 63 Gy or more. They confirmed their results in a multivariate analysis and calculated an improvement of 3 % per Gy in the 5-year local control and overall survival rate. However, possible improvements in local control by dose-escalation have to be weighed against toxicity and functional outcome. For example Mundt et al. [27] observed a severe complication rate of 0 % for doses < 63 Gy compared to 23 % with doses exceeding 63 Gy in grossly resected soft tissue sarcomas. Stinson et al. [28] also described significantly worse functional outcomes for doses of more than 63 Gy. Kepka et al. [8] described a major complication rate of 8 % for doses less or equal to 68 Gy compared to 27 % for doses exceeding 68 Gy in unresectable soft tissue sarcomas, and Slater et al. [26] observed 5 of 6 severe complications in patients treated with 70 Gy or more. Although we could not establish any dose-effect relationship in our series due to the small number of patients, we continue to use our margin-dependent dose concept, attempting 60 Gy after resection with free margins, 66 Gy in cases with microscopic residual disease and 70–72 Gy if gross residual disease is present using the dose constraints for organs at risk established in head and neck cancer [29–34].

Clearly our study has some limitations, namely its retrospective nature, the small sample size and the relatively short follow up. Nevertheless it shows very promising results in a rare patient group and therefore adds valuable information to the existing literature.

## Conclusion

In summary, excellent local control and overall survival rates with acceptable acute and late toxicities can be achieved with postoperative or definitive radiation therapy. Achievement of local control seems of paramount importance because of the close relationship between local control and survival in head and neck sarcomas. Therefore radiation therapy should be added to surgery at least in high grade sarcomas or patients with close or positive margins after surgery despite the absence of randomized trials specifically addressing this rare entity. Although we could not establish a dose-effect relationship in our series, we continue to use our margin-dependent dose concept, attempting 60 Gy after resection with free margins, 66 Gy in cases with microscopic residual disease and 70–72 Gy if gross residual disease is present.

## Abbreviations

Cm: Centimetre
UICC: Union Internationale Contre Le Cancer
FNCLCC: Fédération Nationale Des Centres De Lutte Contre Le Cancer
MFH: Malignant fibrous histiocytoma/undifferentiated pleomorphic sarcoma
CTCAE: Common toxicity criteria adverse events
Gy: Gray
3D-CRT: Three-dimensional conformal radiation therapy
IMRT: Intensity-modulated radiation therapy
n: Number
DFSP: Dermatofibrosarcoma protuberans
PTV: Planning target volume
CT: Computed tomography
MRI: Magnetic resonance imaging
LC: Local control
FFTF: Freedom from treatment failure
OS: Overall survival
LR: Local recurrence
DSM: Distant metastasis
RT: Radiation therapy

## References

[1] Jemal A, Siegel R, Xu J, Ward E (2010). Cancer statistics, 2010. CA Cancer J Clin.

[2] Kraus DH, Dubner S, Harrison LB, Strong EW, Hajdu SI, Kher U (1994). Prognostic factors for recurrence and survival in head and neck soft tissue sarcomas. Cancer.

[3] Bentz BG, Singh B, Woodruff J, Brennan M, Shah JP, Kraus D (2004). Head and neck soft tissue sarcomas: a multivariate analysis of outcomes. Ann Surg Oncol..

[4] Roeder F (2015). Neoadjuvant/adjuvant radiation therapy in soft tissue sarcomas. Journal Onkologie.

[5] Mattavelli D, Miceli R, Radaelli S, Mattavelli F, Cantu G, Barisella M (2013). Head and neck soft tissue sarcomas: prognostic factors and outcome in a series of patients treated at a single institution. Ann Oncol.

[6] Kraus D (2002). Sarcomas of the head and neck. Curr Oncol Rep.

[7] Yang JC, Chang AE, Baker AR, Sindelar WF, Danforth DN, Topalian SL (1998). Randomized prospective study of the benefit of adjuvant radiation therapy in the treatment of soft tissue sarcomas of the extremity. J Clin Oncol.

[8] Kepka L, DeLaney TF, Suit HD, Goldberg SI (2005). Results of radiation therapy for unresected soft-tissue sarcomas. Int J Radiat Oncol Biol Phys.

[9] Le Vay J, O`Sullivan B, Catton C, Cummings B, Fornasier V, Gullane P (1994). An assessment of prognostic factors in soft-tissue sarcoma of the head and neck. Arch Otolaryngol Head Neck Surg.

[10] Willers H, Hug EB, Spiro IJ, Efird JT, Rosenberg AE, Wang CC (1995). Adult soft tissue sarcomas of the head and neck treated by radiation and surgery or radiation alone: patterns of failure and prognostic factors. Int J Radiat Oncol Biol Phys.

[11] Greager JA, Patel MK, Briele HA, Walker MJ, DasGupta TK (1985). Soft tissue sarcomas of the adult head and neck. Cancer.

[12] Weber RS, Benjamin RS, Peters LJ, Ro JY, Achon O, Goepfert H (1986). Soft tissue sarcomas of the head and neck in adolescents and adults. Am J Surg.

[13] Tran LM, Mark R, Meier R, Calcaterra TC, Parker RG (1992). Sarcoma of the Head and Neck: Prognostic Factors and Treatment Strategies. Cancer.

[14] Dudhat SB, Mistry RC, Varughese T, Fakih AR, Chinoy RF (2000). Prognostic factors in head and neck sarcomas. Cancer.

[15] Le QT, Fu KK, Kroll S, Fitts L, Massulo V, Ferrell L (1997). Prognostic factors in adult soft tissue sarcomas of the head and neck. Int J Radiat Oncol Biol Phys.

[16] Eeles RA, Fisher C, A´Hern RP, Robinson M, Rhys-Evans P, Henk JM (1993). Head and neck sarcomas: prognostic factors and implications for treatment. Br J Cancer.

[17] Dijkstra MD, Balm AJ, Coevorden FV, Gregor RT, Hart AA, Hilgers FJ (1996). Suvrival of adult patients with head and neck soft tissue sarcomas. Clin Otolaryngol Allied Sci.

[18] Trifiletti D, Amdur RJ, Dagan R, Indelicato DJ, Mendenhall WM, Kirwan JM (2012). Radiotherapy following gross ttal resection of adult soft tissue sarcoma of the head and neck. Pract Radiat Oncol.

[19] Roeder F, Lehner B, Schmitt T, Kasper B, Egerer G, Sedlaczek O (2014). Excellent local control with IOERT and postoperative EBRT in high grade extremity sarcoma: results from a subgroup analysis of a prospective trial. BMC Cancer.

[20] Jebsen NL, Trovik CS, Bauer HC, Rydholm A, Monge OR, Sundby Hall K (2008). Radiotherapy to improve local control regardless of surgical margin and malignancy grade in extremity and trunk wall soft tissue sarcoma: a Scandinavian sarcoma group study. Int J Radiat Oncol Biol Phys.

[21] Roeder F, Ulrich A, Habl G, Uhl M, Saleh-Ebrahimi L, Huber PE (2014). Clinical phase I/II trial to investigate preoperative dose-escalated intensity-modulated radiation therapy (IMRT) and intraoperative radiation therapy (IORT) in patients with retroperitoneal soft tissue sarcoma: interim analysis. BMC Cancer.

[22] Jensen AD, Uhl M, Chaudri N, Herfarth KK, Debus J, Roeder F (2015). Carbon ion irradiation in the treatment of grossly incomplete or unresectable malignant peripheral nerve sheath tumors: acute toxicity and preliminary outcome. Radiat Oncol.

[23] Zagars GK, Ballo MT (2003). Significance of dose in postoperative radiotherapy for soft tissue sarcoma. Int J Radiat Oncol Biol Phys.

[24] Fein DA, Lee WR, Lanciano RM, Corn BW, Herbert SH, Hanlon AL (1995). Management of extremity soft tissue sarcoma with limb-sparing surgery and postoperative irradiation: do total dose, overall treatment time, and the surgery-radiotherapy impact on local control ?. Int J Radiat Oncol Biol Phys.

[25] Tepper JE, Suit HD (1985). Radiation therapy alone for sarcoma of soft tissue. Cancer.

[26] Slater JD, McNeese MD, Peters LJ (1986). Radiation therapy for unresectable soft tissue sarcomas. Int J Radiat Oncol Biol Phys.

[27] Mundt AJ, Awan A, Sibley GS, Simon M, Rubin SJ, Samuels B (1995). Conservative surgery and adjuvant radiation therapy in the management of adult soft tissue sarcoma of the extremities: clinical and radiobiological results. Int J Radiat Oncol Biol Phys.

[28] Stinson SF, DeLaney TF, Greenberg J, Yang JC, Lampert MH, Hicks JE (1991). Acute and long-term effects on limb function of combined modality limb sparing therapy for extremity soft tissue sarcoma. Int J Radiat Oncol Biol Phys.

[29] Marks LB, Yorke ED, Jackson A, Te Haken RK, Constine LS, Eisbruch A (2010). Use of normal tissue complication probability models in the clinic. Int J Radiat Oncol Biol Phys.

[30] Studer G, Linsenmaier C, Riesterer O, Najafi Y, Brown M, Yousefi B (2013). Late term tolerance in head and neck cancer patients irradiated in the IMRT era. Radiat Oncol.

[31] Merlotti A, Alterio D, Vigna-Taglianti R, Muraglia A, Lastrucci L, Manzo R (2014). Technical guidelines for head and neck cancer IMRT on behalf of the Italian association of radiation oncology – head and neck working group. Radiat Oncol.

[32] Castelli J, Simon A, Louvel G, Henry O, Chajon E, Nassef M (2015). Impact of head and neck cancer adaptive radiotherapy to spare the parotid glands and decrease the risk of xerostomia. Radiat Oncol.

[33] Gupta T, Hotwani C, Kannan S, Master Z, Rangarajan V, Murthy V (2015). Prospective longitudinal assessment of parotid gland function using dynamic quantitative pertechnate scintigraphy and estimation of dose–response relationship of parotid.sparing radiotherapy in head-neck cancers. Radiat Oncol.

[34] Thomas TO, Refaat T, Choi M, Bacchus I, Sachdey S, Rademaker AW (2015). Brachial plexus dose tolerance in head and neck cancer patients treated with sequential intensity modulated radiation therapy. Radiat Oncol.

